# Elucidation of Antioxidant Compounds in Moroccan *Chamaerops humilis* L. Fruits by GC–MS and HPLC–MS Techniques

**DOI:** 10.3390/molecules26092710

**Published:** 2021-05-05

**Authors:** Hafssa El Cadi, Hajar El Bouzidi, Ginane Selama, Btissam Ramdan, Yassine Oulad El Majdoub, Filippo Alibrando, Katia Arena, Miguel Palma Lovillo, Jamal Brigui, Luigi Mondello, Francesco Cacciola, Tania M. G. Salerno

**Affiliations:** 1 Laboratory of Valorization of Resources and Chemical Engineering, Department of Chemistry, Abdelmalek Essaadi University, Tangier 90000, Morocco; hafssa.elcadi@yahoo.fr (H.E.C.); hajarelbouzidi1995@gmail.com (H.E.B.); jamalbrigui@yahoo.fr (J.B.); 2 Laboratory of Biochemistry and Molecular Genetics, Abdelmalek Essaadi University, Tangier 90000, Morocco; ginane.selama@gmail.com; 3 Department of Biology, Laboratory of Biotechnology and Valorization of Natural Resources, Faculty of Science, University Ibn Zohr, Agadir 80000, Morocco; ramdanbtissam8@gmail.com; 4 Department of Chemical, Biological, Pharmaceutical and Environmental Sciences, University of Messina, 98168 Messina, Italy; youladelmajdoub@unime.it (Y.O.E.M.); arenak@unime.it (K.A.); lmondello@unime.it (L.M.); 5 Chromaleont s.r.l., c/o Department of Chemical, Biological, Pharmaceutical and Environmental Sciences, University of Messina, 98168 Messina, Italy; filippo.alibrando@chromaleont.it; 6 Department of Analytical Chemistry, Faculty of Sciences, Agrifood Campus of International Excellence (ceiA3), University of Cadiz, IVAGRO, 11510 Cadiz, Spain; miguel.palma@uca.es; 7 Department of Sciences and Technologies for Human and Environment, University Campus Bio-Medico of Rome, 00128 Rome, Italy; 8 BeSep s.r.l., c/o Department of Chemical, Biological, Pharmaceutical and Environmental Sciences, University of Messina, 98168 Messina, Italy; tania.salerno@besep.it; 9 Department of Biomedical, Dental, Morphological and Functional Imaging Sciences, University of Messina, 98125 Messina, Italy

**Keywords:** Arecaceae, polyphenols, volatile content, antioxidant activity, liquid chromatography

## Abstract

The aim of this study was to characterize the phytochemical content as well as the antioxidant ability of the Moroccan species *Chamaerops humilis* L. Besides crude ethanolic extract, two extracts obtained by sonication using two solvents with increased polarity, namely ethyl acetate (EtOAc) and methanol-water (MeOH-H_2_O) 80:20 (*v*/*v*), were investigated by both spectroscopy and chromatography methods. Between the two extracts, the MeOH-H_2_O one showed the highest total polyphenolic content equal to 32.7 ± 0.1 mg GAE/g DM with respect to the EtOAc extract (3.6 ± 0.5 mg GAE/g DM). Concerning the antioxidant activity of the two extracts, the EtOAc one yielded the highest value (1.9 ± 0.1 mg/mL) with respect to MeOH-H2O (0.4 ± 0.1 mg/mL). The *C. humilis* *n*-hexane fraction, analyzed by GC–MS, exhibited 69 compounds belonging to different chemical classes, with *n*-Hexadecanoic acid as a major compound (21.75%), whereas the polyphenolic profile, elucidated by HPLC–PDA/MS, led to the identification of a total of sixteen and thirteen different compounds in both EtOAc (major component: ferulic acid: 104.7 ± 2.52 µg/g) and MeOH-H_2_O extracts (major component: chlorogenic acid: 45.4 ± 1.59 µg/g), respectively. The attained results clearly highlight the potential of *C. humilis* as an important source of bioactive components, making it a valuable candidate to be advantageously added to the daily diet. Furthermore, this study provides the scientific basis for the exploitation of the Doum in the food, pharmaceutical and nutraceutical industries.

## 1. Introduction

The Moroccan wild palm tree (*Chamaerops humilis* L.), widely called “Doum”, is found in six cities of the eastern region of Morocco, namely Oujda, Berkane, Ahfir, Saidia, Nador and Jerrada [1], and represents 7.74% of the total number of Moroccan palm trees [2].

Such a species is cultivated in many Mediterranean countries as an ornament, considering its robustness and decorative features.

Some components of this plant have been used as food as an important source of nutritional energy [3], or in traditional medicine. The husks are eaten in Southern Spain, the fruits in Morocco and the young suckers in Italy. Leaf extracts of *Chamaerops humilis* L. (*C. humilis*) have been commonly used for the treatment of diabetes, digestive disorders, spasms, tone and gastrointestinal disorders [4,5]. Moreover, their fruits have astringent properties thanks to their tannin content, even though, in Morocco, they have been rarely consumed due to their bitter taste [4].

Other studies have shown the beneficial effects of these fruits against hyperlipidemia in an animal model of obesity and hyperglycemia [6]. Thanks to their sedative action, they have been also used to treat insomnia, cough attacks and bronchitis [7]; also, the “Doum” has shown anti-inflammatory, anabolic, antiseptic, urinary, antilithic and diuretic activities [4,7,8]. Leaf extracts have also been reported to possess antioxidant activity and the ability to inhibit lipoxygenase [9,10].

The phytochemical properties of *C. humilis* are so far only little characterized. The analysis of the grain’s oil showed higher levels of oleic and linoleic acids than other seed oils, as well as a significant amount of tocopherols and tocotrienols [11].

Several biologically important secondary metabolites such as flavonoids, phenols, saponins, gallic tannins and terpenoids have been detected in the leaves and fruits of *C. humilis* L., which may explain the pharmacological effects mentioned above [4,7,9,12].

With regard to flavonoids, they have been previously reported as constituents of the Arecaceae family of plants, even though the literature lacks detailed information on the phytochemical composition of *C. humilis.* Further, no work has been so far devoted to the analysis of the volatile content of such a species.

The aim of this work was to determine the volatile and polyphenolic content of Moroccan Doum fruits (*C. humilis* L.) by GC–MS and HPLC–PDA/MS. In addition, the evaluation of the physico-chemical properties, and the antioxidant activities of the fruit extracts, was performed as well.

This study represents an effort to provide more reliable information about the antioxidant and beneficial health properties of such a species in order to promote its use in different food, pharmaceutical and supplement industries.

## 2. Results and Discussion

### 2.1. Physico-Chemical Parameters

Table 1 reports the physico-chemical parameters for the *C. humilis* fruit under investigation.

The percentage of dry matter attained was equal to 69.6 ± 0.5, approximately indicating the presence of 30.4% water in these fruits. The latter value is twice as high than the one reported by Bouhafsoun et al. (17.4 ± 0.12%) [2]. On the other hand, another study showed a higher value (79.6 ± 0.04%) in *Butia odorata*, which belongs to the same family (Arecaceae) [13].

The ash content revealed interesting amounts of minerals (3.0 ± 0.3). Such a value coincides with the mean value of ash content (2.4 to 5.0%) recommended by FAO [14], even though it is lower than that recently reported for the Algerian species (4.2 ± 0.7%) [2].

Concerning the pH measurement, a value of 3 ± 0.06 was attained. This value is lower than the one found by Bouhafsoun et al. (5.0 ± 0.0) [2] and, in general, other species belonging to the Arecaceae family, e.g., date palm (*Phoenix dactylifera* L.) (5.3 ± 0.0) [15] and doum palm (*Hyphaene thebaica)* (4.8 ± 0.0) [16].

The titratable acidity of *C. humilis* L. fruit revealed a percentage of 1.5 ± 0.3%. This value is slightly different from Algerian fruits (0.2 ± 0.0%) [2], but similar to other species, e.g., *Hyphaene thebaica* (0.22%) [16].

The TSS results showed a mean value of 15.2 ± 0.7%. Similar values were found in *Butia odorata* fruits (13.1–14.6%) [17], despite Ferrão et al. (2013) revealing, for the same species, a value of 9.5 ± 0.0% [13]. These results are not in agreement with other studies where values reported were 2.4% in leaves and rachis and 4% in fruits [2]. This can be directly related to the sugar content of the fruit samples, which have higher sugar content than other parts of *C. humilis* L. [2].

The S/A ratio was 10.3 ± 0.5%. Such a ratio is an important biochemical parameter that influences the taste and acceptability of the fruits. The high values of this ratio indicate good technological properties and consumer acceptance of these fruits [18,19]. The result achieved in this study falls within the range found for *Butia odorata* fruits (4.42–14.20%) [13]. On the other hand, the S/A ratio values of the *C. humulis* L. fruits investigated in this work showed higher values compared to those of *B. capitata* reported in the literature, 4.7–5.8% [20].

Results of RS and TS were equal to 18.1 ± 0.7% and 23.7 ± 0.9%, respectively. Vitamin C contents in Doum extracts were determined to be 31.5 ± 0.5 mg/g, which is slightly higher than other research (20.1 ± 0.5 mg/g) [21].

The refractive index values for the *C. humilis* L. in each extract were 1.3 ± 0.0 and 1.34 ± 0.0 for ethyl acetate (EtOAc) and methanol–water (MeOH-H_2_O), respectively. The ANOVA test (*p* > 0.05) showed that the difference between the fruits in IR was not significant.

With regard to lipid and protein contents, values of 0.7 ± 0.0% and 5.3 ± 1.5% were attained, respectively. A value of 0.6 ± 0.0 mg/g was attained for the MeOH-H_2_O extract, whereas they were absent in the EtOAc fraction. The low levels of protein content can be caused by the ultrasonic extraction, which leads to protein denaturation, as proven by some researchers [22].

### 2.2. Phytochemical Screening

The phytochemical screening of *C. humilis* was carried out, for the first time for a Moroccan species. The phytochemical tests revealed the presence of different chemical families, distributed for the studied species according to the solvent concentration used. Anthocyanins were not detected in any of the samples investigated. In the EtOAc extract, unsaturated sterols, terpenes and glycosides, which were absent in the crude extract, were revealed. On the other hand, in the crude extract, catechic tannins, anthracenosides, sterols and steroids were detected in high concentrations.

In the literature, the phytochemical properties of *C. humilis* are not well characterized, although several studies have reported the presence of tannins, flavonoids, saponins, sterols and terpenoids [10]. These results are similar to those found in samples from Algeria [23]. Notably, saponosides, responsible for many pharmacological properties, e.g., anti-inflammatory [24,25], were also detected in the extract of *C. humilis*. From the results achieved, such a species does contain important phytochemical constituents that may contribute to its anti-inflammatory and antioxidant activities (Table 2).

### 2.3. Phytochemical Content and Antioxidant Ability

The spectrophotometric assays showed an important amount of polyphenols. Comparing the two extracts, the MeOH-H_2_O one showed the highest total polyphenolic (TPP) content, equal to 32.7 ± 0.1 mg GAE/g DM, with respect to the EtOAc extract, 3.6 ± 0.5 mg GAE/g DM (Table 3). The same considerations can be made for the total flavonoid (TFv) and total tannin (TT) contents.

Statistical analysis (ANOVA) showed that there was a highly significant difference in results (*p* < 0.001) between the different solvent concentrations, thus indicating an effect of the solvent concentration on the extraction of these compounds [26].

Concerning the antioxidant activity of the two fractions (1.9 ± 0.1 mg/mL and 0.4 ± 0.1 mg/mL, respectively, for EtOAc and MeOH-H_2_O), the values attained are higher than those found by other authors, e.g., Belhaoues et al. (2017) [27] and Gonçalves et al. (2018) [10] (0.12 mg/mL and 0.081 mg/mL). Another two studies obtained from a methanolic extract of *C. humilis* reported IC_50_ values of 0.024 mg/mL [28] and 0.455 mg/mL [29]. Our findings are in agreement with previously reported papers on different species [26,30].

### 2.4. GC–MS Analyses

**Figure 1:**
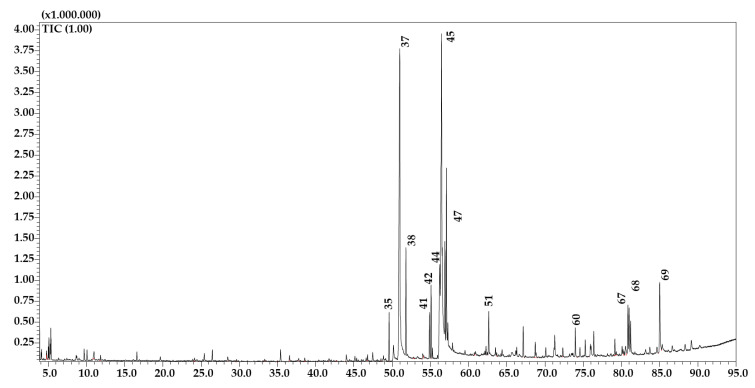
GC–MS profile of the *n*-hexane fraction of *C. humilis.* Main peaks are labeled. Peak assignment as in Table 4.

The *C. humilis n*-hexane fraction exhibited 69 compounds belonging to different chemical classes (Figure 1). Similarity ranged from 87% to 96%. The main volatile compound was represented by *n*-Hexadecanoic acid (21.75%), followed by oleic acid (14.66%) (Table 4). Such findings are consistent with other *C. humilis* works [31,32].

### 2.5. HPLC–PDA/MS Analyses

**Figure 2:**
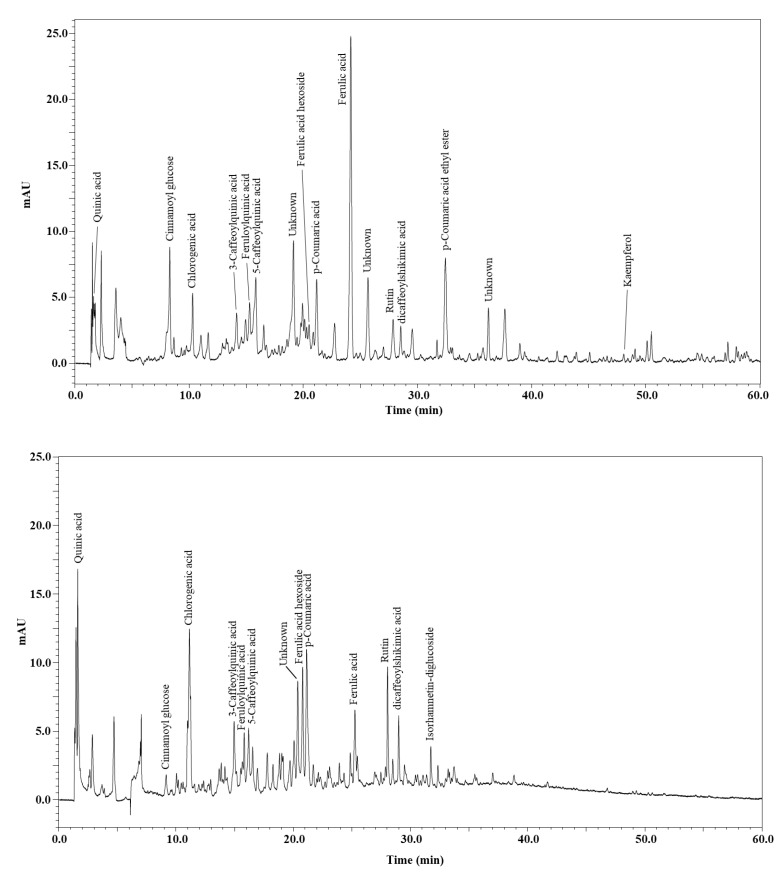
HPLC–PDA polyphenolic profile of the EtOAc (top) and MeOH-H_2_O extracts of *C. humilis.*

The analysis of the polyphenolic profile, achieved by HPLC–PDA of the EtOAc and MeOH-H_2_O extracts of *C. humilis*, is reported in Figure 2. A total of sixteen and thirteen different polyphenolic compounds were detected in both extracts, respectively. Tentative identification was based on combined data coming from retention times, PDA, MS and standard co-injection, when available (thirteen in EtOAc vs. twelve in MeOH-H_2_O extracts (Table 5 and Table 6)). Interestingly, the totality of the polyphenolic compounds in both extracts belong to the hydroxycinnamic acids class, whereas only two flavonols were identified in both extracts. Most of the compounds were already reported as constituents of fruits of botanical species, belonging to the same family, e.g., ferulic acid, feruloylquinic acid, ferulic acid hexoside [33], *p*-Coumaric acid, dicaffeoylshikimic acid and isorhamnetin-diglucoside [34]. Notably, 3-Caffeoylquinic acid and 3-Caffeoylquinic acid were reported as constituents of leaf extracts of *C. humilis* [10], whereas quinic acid, *p*-Coumaric, rutin and kaempferol were found in the fruits of the same species [23]. Cinnamoyl glucose and *p*-Coumaric acid ethyl ester are here reported for the first time.

The quantification was determined for three repetitions of different extracts of the same sample. As far as quantification is concerned (Table 7), ferulic acid in the EtOAc extract turned out to be the most abundant one (104.7 µg/g), followed by 5-Caffeoylquinic acid (36.5 µg/g). On the other hand, in the MeOH-H_2_O extract, chlorogenic acid (45.4 µg/g) was predominant, along with quinic acid (37.0 µg/g).

In total, 276.7 µg/g and 262.2 µg/g of polyphenolic compounds for the EtOAc and MeOH-H_2_O extracts of *C. humilis*, respectively, were attained. Such results are comparable with other Moroccan fruits, e.g., *Ziziphus lotus*, at least for the EtOAc extract (298.5 µg/g) [26].

## 3. Materials and Methods

### 3.1. Samples and Sample Extraction

*Chamaerops humilis* L. fruits were harvested in Tangier-Tetouan-Al Hoceima, an area located in the extreme north-west of Morocco. The samples were collected for 4 months (May, June, July and August 2018). All of the harvest areas were between the longitudes 5°94’84106 and the latitudes 35°44’701. The fruit harvesting was carried out at their physiological maturity in the early morning, transported in well-closed boxes and stored at −10 °C in the Materials and Resources Valorization Laboratory, Faculty of Sciences and Technology of Tangier. The extraction method employed was previously described by El Cadi et al. (2020) [26]. Briefly, 5 g of lyophilized powder underwent a defatting step by adding three times 50 mL of *n*-hexane; afterwards, it was dried and homogenized with 50 mL of two solvents with increased polarity, namely EtOAc and MeOH-H_2_O 80:20 (*v*/*v*). Each fraction was extracted by using an ultrasound bath (130 kHz) for 45 min. After centrifugation at 5000 g for 5 min, the supernatant was filtered through a paper filter, dried, reconstituted with MeOH-H_2_O and then filtered through a 0.45 μm Acrodisc nylon membrane (Merck Life Science, Merck KGaA, Darmstadt, Germany) prior to HPLC–PDA-ESI/MS analysis.

### 3.2. Chemical Reagents and Solvents

Folin-Ciocalteu phenol reagent was obtained from Fluka. Standards (gallic acid, caffeic acid, cinnamic acid, ferulic acid, coumarin, rutin and kaempferol) were obtained from Merck Life Science (Merck KGaA, Darmstadt, Germany). In addition, 2,2-diphenyl-1-picrylhydrazyl (DPPH) and butylated hydroxytoluene (BHT) were purchased from Sigma (St. Louis, MO, USA). LC-MS grade methanol, acetonitrile, acetic acid, EtOAc, acetone and water were purchased from Merck Life Science (Merck KGaA, Darmstadt, Germany). All of the other chemicals were of analytical grade and obtained from Sigma (St. Louis, MO, USA).

### 3.3. Physico-Chemical Analyses and Phytochemical Screening

Physico-chemical analyses and phytochemical screening were carried out according to a previously published work [26].

### 3.4. Analysis and Quantification of Phenolic Contents

TPP content was estimated using Folin-Ciocalteu method [35] and was expressed as mg of gallic acid (GAE)/g of dry mass (DM). TFv content was expressed as mg of quercetin (QE)/g of dry mass (DM) and quantified according to the method of Zhishen et al. [36]. TT content was determined by the vanillin method of Julkunen-Tiitto and expressed as mg (+)-catechin/g DW [37].

### 3.5. Determination of Antioxidant Activity

The DPPH method followed the method described by Braca et al. [38]. Butylated hydroxytoluene (BHT) was used as a positive control and the DPPH radical scavenging activity was calculated according to the equation:

*DPPH radical scavenging activity: I *(%) = (*A blank* − *A sample*) / *A blank* × 100 (1)

The IC_50_ of the DPPH radical was calculated from linear regression (%DPPH remaining radical versus sample concentration).

### 3.6. GC–MS

GC analyses of the volatile fraction were performed on a GC–MS-QP2020 system (Shimadzu, Kyoto, Japan) with an “AOC-20i” system auto-injector. The analyses were realized on an SLB-5ms column (30 m in length × 0.25 mm in diameter × 0.25 µm in thickness of film, Merck KGaA). The initial temperature was set at 50 °C, and afterwards increased up to 350 °C (increase rate: 3 °C/min; holding time: 5 min).

GC–MS parameters were as follows: injection temperature: 280 °C; injection volume: 1.0 µL (split ratio: 10:1); pure helium gas (99.9%); linear velocity: 30.0 cm/s; inlet pressure: 26.7 KPa; EI source temperature: 220 °C; interface temperature: 250 °C. The acquisition of MS spectra was realized in full scan mode, in the mass range of 40–660 m/z, with an event time of 0.2 s.

Relative quantity of the chemical compounds present in each sample was expressed as a percentage based on peak area produced in the GC chromatogram.

Compounds were identified by using the “FFNSC 4.0” (Shimadzu Europa GmbH, Duisburg, Germany) and “W11N17” (Wiley11-Nist17, Wiley, Hoboken, NJ, USA; Mass Finder 3). Each compound was identified applying a MS similarity match and an LRI filter. Linear retention indices (LRI) were calculated by using a C7–C40 saturated alkanes reference mixture (49452-U, MerckKGaA).

Data files were collected and processed by using “GCMS Solution” software, ver. 4.50 (Shimadzu, Kyoto, Japan) [26].

### 3.7. HPLC–PDA/ESI-MS

LC analyses were performed on a Shimadzu liquid chromatography system (Kyoto, Japan), consisting of a CBM-20A controller, two LC-30AD dual-plunger parallel-flow pumps, a DGU-20A5R degasser, a CTO-20AC column oven, a SIL-30AC autosampler, an SPD-M30A photo diode array detector and an LCMS-8050 triple quadrupole mass spectrometer, through an ESI source (Shimadzu, Kyoto, Japan).

Chromatographic separations were attained on 150 × 4.6 mm; 2.7 µm Ascentis Express RP C18 columns (Merck Life Science, Merck KGaA, Darmstadt, Germany). The mobile phase was composed of two solvents: water/acetic acid (99.85/0.15 *v*/*v*, solvent A) and acetonitrile/acetic acid (99.85/0.15 *v*/*v*, solvent B). The flow rate was set at 1 mL/min under gradient elution: 0–5 min, 5% B, 5–15 min, 10% B, 15–30 min, 20% B, 30–60 min, 50% B, 60 min, 100% B. PDA detection was: λ = 200–400 nm (λ = 280 nm) (sampling frequency: 40.0 Hz, time constant: 0.08 s). MS conditions were as follows: scan range and the scan speed were set at *m*/*z* 100–800 and 2500 amu sec ^−1^, respectively, event time: 0.3 sec, nebulizing gas (N_2_) flow rate: 1.5 L min^−1^, drying gas (N_2_) flow rate: 15 L min^−1^, interface temperature: 350 °C, heat block temperature: 300 °C, DL (desolvation line) temperature: 300 °C, DL voltage: 1 V, interface voltage: −4.5 kV [26].

### 3.8. Statistical Analysis

The experiments were carried out in triplicate and the results were expressed as the average of the three measurements ± SD. The comparison of means between groups was performed with one-way analysis of variance (ANOVA) followed by a Tukey test. Differences were considered significant when *p* < 0.05 (Microsoft ^®^ Office, Santa Rosa, California, CA, USA). 

## 4. Conclusions

The present study aimed to elucidate the bioactive content of *Chamaerops humilis* L. fruits. Considering the two extracts tested, in terms of the antioxidant activity, the EtOAc one turned out to be the most active with respect to the MeOH-H_2_O. A total of 69 compounds belonging to different chemical classes were positively identified by GC coupled to MS, whereas sixteen and thirteen polyphenolic compounds were detected by HPLC–PDA/MS in both EtOAc and MeOH-H_2_O extracts, respectively. Such results demonstrate that this fruit can be used for industrial applications in food preparations. In addition, the data attained emphasize an interesting functional composition of the *Chamaerops humilis* L. fruits, which could be considered a valuable new co-product with commercial importance in the food industry.

## Figures and Tables

**Table 1:** Physico-chemical parameters of *C. humilis* fruit samples. The results are expressed as mean ± standard deviation.

Fruit	*Crude Extract*	*Solvent Fractions*	
		EtOAc	MeOH-H_2_O
**pH**	3.0 ± 0.06	−	−
**Acidity**	1.5 ± 0.28	−	−
**RI**	1.4 ± 0.10	1.3 ± 0.00	1.3 ± 0.00
**TSS**	15.2 ± 0.68	0.4 ± 0.01	3.0 ± 0.01
**S/A**	10.3 ± 0.5	−	−
**DM (%)**	69.5 ± 0.51	−	−
**Ash (%)**	3.0 ± 0.31	−	−
**TS (%)**	23.7 ± 0.86	6.4 ± 0.05	4.6 ± 0.10
**RS (%)**	18.1 ± 0.72	−	−
**Lipids** **(mg/g)**	0.70 ± 0.05	−	−
**Proteins** **(mg/g)**	5.33 ± 1.5	−	0.6 ± 0.01
**Vitamin C (mg/g)**	31.4 ± 0.53	13.6 ± 0.45	30 ± 0.28

RI: refractive index; TSS: total soluble solid (°Brix); DM: dry matter; S/A: sugar/acidity; TS: total sugars; RS: reducing sugars.

**Table 2:** Phytochemical screening of *C. humilis* samples.

Compounds Group/Solvent of Extraction		Crude Extract	EtOAc	MeOH-H_2_O
**Alkaloids**		+	+	±
**Polyphenols**	Flavonoids	B	B	A++
	Tannins	+	+	+
	Anthocyanins	−	−	−
	Catechic tannins	++	−	+
	Gallic tannins	−	−	+
	Coumarins	+	+	+
	Anthracenosides	++	−	−
	Anthraquinones	+	−	−
	Anthracenosides and Anthocyanosides	+	−	−
**Steroids**	Saponosides	++	-	−
	Unsaturated Sterols/Terpenes	−	+	−
	Sterols and Steroids	++	−	−
**Sugars**	Starch	+	−	−
	Deoxysugars	+	−	−
	Glycosides	−	+	±
	Mucilages	+	−	+

A: Flavones; B: Isoflavones; ++: Abondant; +: Present; −: Absent.

**Table 3:** TPP, TFv and TT content in *C. humilis* solvent fractions.

Extract	TPP	TFv	TT	IC_50_
**EtOAc**	3.6 ± 0.5	6.5 ± 0.1	6.2 ± 0.5	1.9 ± 0.1
**MeOH-H_2_O**	32.7 ± 0.1	11.1 ± 0.45	54.3 ± 0.8	0.4 ± 0.1

**Table 4:** List of compounds identified in the *n*-hexane fraction of *C. humilis* by GC–MS.

No.	Compound	LRI (lib)	LRI (exp)	Similarity	Area(%)	Library
1	*n*-Hexanol	867	867	90	0.04	FFNSC 4.0
2	Acetonylacetone	923	925	90	0.11	FFNSC 4.0
3	*n*-Hexanoic acid	997	977	96	0.31	FFNSC 4.0
4	*n*-Nonanal	1107	1106	96	0.27	FFNSC 4.0
5	*n*-Octanoic acid	1192	1171	94	0.19	FFNSC 4.0
6	*n*-Decanal	1208	1207	91	0.06	FFNSC 4.0
7	(2E)-Decenal	1265	1264	92	0.06	FFNSC 4.0
8	Nonanoic acid	1289	1269	92	0.13	FFNSC 4.0
9	(2E,4E)-Decadienal	1322	1296	93	0.41	FFNSC 4.0
10	*n*-Decanoic acid	1398	1366	93	0.15	FFNSC 4.0
11	ethyl-Decanoate	1399	1395	93	0.08	FFNSC 4.0
12	(E)-, β-Ionone	1482	1482	87	0.07	FFNSC 4.0
13	methyl-Dodecanoate	1527	1524	88	0.03	FFNSC 4.0
14	5,6,7,7a-tetrahydro-4,4,7a-trimethyl-,(R)-2(4H)-Benzofuranone	1532	1533	90	0.46	W11N17
15	*n*-Dodecanoic acid	1581	1563	94	0.18	FFNSC 4.0
16	ethyl-Dodecanoate	1598	1594	89	0.11	FFNSC 4.0
17	*n*-Hexadecane	1600	1600	87	0.03	FFNSC 4.0
18	*n*-Tetradecanal	1614	1614	91	0.13	FFNSC 4.0
19	1.1’-oxybis-Octane	1657	1663	88	0.10	W11N17
20	*n*-Heptadecane	1700	1700	90	0.17	FFNSC 4.0
21	2-Pentadecanol	1710	1707	92	0.05	W11N17
22	Pentadecanal	1717	1716	90	0.08	W11N17
23	methyl-Tetradecanoate	1727	1725	87	0.03	FFNSC 4.0
24	*n*-Tetradecanoic acid	1773	1762	87	0.23	FFNSC 4.0
25	ethyl-Tetradecanoate	1794	1793	93	0.22	FFNSC 4.0
26	*n*-Octadecane	1800	1800	91	0.09	FFNSC 4.0
27	Hexadecanal	1820	1818	93	0.09	W11N17
28	Pentadecanoic acid, methyl ester	1824	1825	88	0.11	W11N17
29	Neophytadiene	1836	1836	92	0.10	FFNSC 4.0
30	Phytone	1841	1842	94	0.19	FFNSC 4.0
31	Pentadecylic acid	1869	1863	90	0.11	FFNSC 4.0
32	ethyl-Pentadecanoate	1893	1893	91	0.09	FFNSC 4.0
33	*n*-Nonadecane	1900	1900	90	0.13	FFNSC 4.0
34	(Z)-9-Hexadecenoic acid, methyl ester	1895	1903	93	0.14	W11N17
35	methyl-Hexadecanoate	1925	1926	95	1.62	FFNSC 4.0
36	Hexadecanolact-16-one	1938	1943	88	0.77	FFNSC 4.0
37	*n*-Hexadecanoic acid	1977	1969	94	21.75	FFNSC 4.0
38	ethyl-Palmitate	1993	1993	96	3.80	FFNSC 4.0
39	Heptadecanoic acid, methyl ester	2028	2026	90	0.06	W11N17
40	Heptadecanoic acid	2080	2064	94	0.33	W11N17
41	methyl-Linoleate	2093	2093	90	1.57	FFNSC 4.0
42	methyl-Oleate	2098	2098	92	2.61	FFNSC 4.0
43	methyl-Octadecanoate	2127	2127	89	0.11	FFNSC 4.0
44	Linoleic acid	2144	2137	92	6.90	FFNSC 4.0
45	Oleic acid	2142	2145	89	14.66	FFNSC 4.0
46	(Z)-Vaccenic acid	2161	2148	92	4.03	W11N17
47	ethyl-Linoleate	2164	2160	92	5.04	FFNSC 4.0
48	(E)-9-Octadecenoic acid ethyl ester	2174	2173	92	2.10	W11N17
49	ethyl-Stearate	2198	2194	91	0.53	FFNSC 4.0
50	*n*-Tricosane	2300	2300	90	0.22	FFNSC 4.0
51	(Z)-9-Octadecenamide	2375	2362	94	1.69	W11N17
52	*n*-Tetracosane	2400	2400	88	0.12	FFNSC 4.0
53	Behenyl alcohol	2493	2495	89	0.19	FFNSC 4.0
54	*n*-Pentacosane	2500	2500	93	0.31	FFNSC 4.0
55	1-Hexacosene	2596	2595	90	0.54	W11N17
56	*n*-Hexacosane	2600	2599	93	0.19	FFNSC 4.0
57	Heptacos-1-ene	2694	2695	89	0.33	W11N17
58	*n*-Heptacosane	2700	2700	92	0.88	FFNSC 4.0
59	*n*-Octacosane	2800	2799	89	0.16	FFNSC 4.0
60	Squalene	2810	2813	94	1.15	FFNSC 4.0
61	Hexacosanal	2833	2840	93	0.41	W11N17
62	*n*-Nonacosane	2900	2900	90	0.50	FFNSC 4.0
63	Octacosanal	3039	3044	95	0.65	W11N17
64	γ-Tocopherol	3055	3053	88	0.15	W11N17
65	*n*-Hentriacontane	3100	3100	92	0.13	FFNSC 4.0
66	Octacosanol	3120	3109	94	0.68	W11N17
67	2-Nonacosanone	3125	3123	91	2.16	W11N17
68	Vitamin E	3130	3131	93	2.07	W11N17
69	γ-Sitosterol	3351	3321	90	4.13	W11N17
	**Tot. identified**				**87.29**	
	**Tot. not identified**				**12.71**	

**Table 5:** Polyphenolic compounds detected in EtOAc extract of *C. humilis* by HPLC–PDA–ESI/MS.

Tentative Identification	t_R_ (min)	Identification Type	λ_MAX_ (nm)	[M-H]^−^	Fragments
**Phenolic Acid and Derivatives**					
**Quinic acid**	1.64	PDA/MS	−	191	−
**Cinnamoyl glucose**	8.31	PDA/MS	258–291	309	−
**Chlorogenic acid**	10.31	PDA/MS	324	353	179
**3-Caffeoylquinic acid**	14.15	PDA/MS	321	353	−
**Feruloylquinic acid**	15.29	PDA/MS	324	367	−
**5-Caffeoylquinic acid**	15.83	PDA/MS	213–324	353	179
**Unknown**	19.13	PDA/MS	282–325	336	−
**Ferulic acid hexoside**	20.51	PDA/MS	214–324	355	191
**p-coumaric acid**	22.74	PDA/MS	288	163	−
**Ferulic acid**	24.16	PDA/MS	216–321	193	−
**Unknown**	25.66	PDA/MS	304	193	−
**dicaffeoylshikimic acid**	28.54	PDA/MS	217–291	497	179
**p-Coumaric acid ethyl ester**	32.46	PDA/MS	247–291	191	−
**Unknown**	36.22	PDA/MS	270	345	263
**Flavonols**					
**Rutin**	27.86	PDA/MS	352	609	−
**Kaempferol**	48.18	PDA/MS	219–369	285	−

**Table 6:** Polyphenolic compounds detected in MeOH-H_2_O extract of *C. humilis* by HPLC–PDA–ESI/MS.

Tentative Identification	t_R_ (min)	Identification Type	λ_MAX_ (nm)	[M-H]^−^	Fragments
**Phenolic Acid and Derivatives**					
**Quinic acid**	1.64	PDA/MS	−	191	−
**Cinnamoyl glucose**	8.31	PDA/MS	258–291	309	−
**Chlorogenic acid**	10.31	PDA/MS	324	353	179
**3-Caffeoylquinic acid**	14.15	PDA/MS	321	353	−
**Feruloylquinic acid**	15.29	PDA/MS	324	367	−
**5-Caffeoylquinic acid**	15.83	PDA/MS	213–324	353	179
**Unknown**	19.13	PDA/MS	282–325	336	−
**Ferulic acid hexoside**	20.51	PDA/MS	214–324	355	191
**p-coumaric acid**	22.74	PDA/MS	288	163	−
**Ferulic acid**	24.16	PDA/MS	216–321	193	−
**Dicaffeoylshikimic acid**	28.54	PDA/MS	217–291	497	179
**Flavonols**					
**Rutin**	27.86	PDA/MS	352	609	−
**Isorhamnetin-diglucoside**	31.75	PDA/MS	353	623	−

**Table 7:** Semi-quantification of polyphenols detected in *C. humilis* fruits in µg/g (*w*/*w*).

Compounds	EtOAc	MeOH-H_2_O	Standard Used for Semi-Quantification
	**Phenolic Acid and Derivatives**		
**Quinic acid**	6.3 ± 0.02	37.0 ± 0.36	Gallic acid
**Cinnamoyl glucose**	8.1 ± 0.40	0.3 ± 0.03	Cinnamic acid
**Chlorogenic acid**	18.8 ± 0.90	45.4 ± 1.59	Caffeic acid
**3-Caffeoylquinic acid**	16.6 ± 0.30	22.4 ± 0.14	Ferulic acid
**Feruloylquinic acid**	26.3 ± 1.02	12.4 ± 0.07	Ferulic acid
**5-Caffeoylquinic acid**	36.5 ± 1.05	20.3 ± 0.62	Caffeic acid
**Ferulic acid hexoside**	12.9 ± 0.82	20.3 ± 0.21	Ferulic acid
**p-coumaric acid**	11.3 ± 0.50	0.4 ± 0.01	Coumarin
**Ferulic acid**	104.7 ± 2.52	20.6 ± 0.9	Ferulic acid
**Dicaffeoylshikimic acid**	7.5 ± 0.10	10.1 ± 0.5	Caffeic acid
***p*-Coumaric acid ethyl ester**	12.7 ± 0.12	−	Coumarin
	**Flavonols**		
**Rutin**	17.7 ± 0.03	60.2 ± 1.9	Rutin
**Isorhamnetin-diglucoside**	−	12.8 ± 0.8	Kaempferol
**Kaempferol**	15.0 ± 0.93	−	Kaempferol

## Data Availability

Data sharing not applicable.

## Sample Availability

Samples of the compounds are not available from the authors.

## References

[1] Tassin C. (2012). Paysages Végétaux du Domaine Méditerranéen: Bassin Méditerranéen, Californie, Chili Central, Afrique du Sud, Australie Méridionale.

[2] Bouhafsoun A., Boukeloua A., Yener I., Diare Mohamed L., Diarra T., Errouane K., Mezmaz R., Temel H., Kaid-Harche M. (2019). Chemical composition and mineral contents of leaflets, rachs and fruits of *Chamaerops humilis* L.. Acad. J. Agric. Res..

[3] Nehdi I.A., Mokbli S., Sbihi H., Tan C.P., Al-Resayes S.I. (2014). *Chamaerops humilis* L. var. argentea André Date Palm Seed Oil: A Potential Dietetic Plant Product: Nutritional value of C. humilis seed oil. J. Food Sci..

[4] Bnouham M., Merhfour F.Z., Ziyyat A., Aziz M., Legssyer A., Mekhfi H. (2010). Antidiabetic effect of some medicinal plants of Oriental Morocco in neonatal non-insulin-dependent diabetes mellitus rats. Hum. Exp. Toxicol..

[5] Hasnaoui O., Bouazza M., Benali O., Thinon M. (2011). Ethno Botanic Study of *Chamaerops humilis* L. Var. argentea Andre (Are-caceae) in Western Algeria. Agric. J..

[6] Gaamoussi F., Israili Z.H., Lyoussi B. (2010). Hypoglycemic and hypolipidemic effects of an aqueous extract of *Chamaerops humilis* leaves in obese, hyperglycemic and hyperlipidemic Meriones shawi rats. Pak. J. Pharm. Sci..

[7] Benmehdi H., Hasnaoui O., Benali O., Salhi F. (2012). Phytochemical investigation of leaves and fruits extracts of *Chamaerops humilis* L.. J. Mater. Environ. Sci..

[8] Blumenthal M., Busse W., Goldberg A., Gruenwald J., Hall T., Riggins C.W., Rister R.S. (1998). The Complete German Commis-sion E Monographs: Therapeutic Guide to Herbal Medicines.

[9] Benahmed-Bouhafsoun A., Djied S., Mouzaz F., Kaid-Harche M. (2013). Phytochemical composition and in vitro antioxidant activity of *Chamaerops humilis* L. extracts. Int. J. Pharm. Pharm. Sci..

[10] Miguel M., Bouchmaaa N., Aazza S., Gaamoussi F., Lyoussi B. (2014). Antioxidant, anti-inflammatory and anti-acetylcholinesterase activities of Moroccan plants. Fresenius Environ. Bull..

[11] Fekar G., Aiboudi M., Bouyazza L. (2015). Composition en acides gras, stérols et tocophérols de l huile végétale non convention-nelle extraite des graines du *Chamaerops humilis* L. du Maroc. Afrique Sci..

[12] Gonçalves S., Medronho J., Moreira E., Grosso C., Andrade P.B., Valentão P., Romano A. (2018). Bioactive properties of *Chamaerops humilis* L.: Antioxidant and enzyme inhibiting activities of extracts from leaves, seeds, pulp and peel. 3 Biotech.

[13] Ferrão T.S., Ferreira D.F., Flores D.W., Bernardi G., Link D., Barin J.S., Wagner R. (2013). Evaluation of composition and quality parameters of jelly palm (*Butia odorata*) fruits from different regions of Southern Brazil. Food Res. Int..

[14] FAO (1988). Street Foods: A Summary of FAO Studies and Other Activities Relating to Street Foods.

[15] Shaba E.Y., Ndamitso M.M., Mathew J.T., Etsunyakpa M.B., Tsado A.N., Muhammad S.S. (2015). Nutritional and anti-nutritional composition of date palm (*Phoenix dactylifera* L.) fruits sold in major markets of Minna Niger State, Nigeria. Afr. J. Pure Appl. Chem..

[16] Aamer R.A. (2016). Characteristics of aqueous doum fruit extract and its utilization in some novel products. Ann. Agric. Sci..

[17] Beskow G.T., Hoffmann J.F., Teixeira A.M., Fachinello J.C., Chaves F.C., Rombaldi C.V. (2015). Bioactive and yield potential of jelly palms (*Butia odorata* Barb. Rodr.). Food Chem..

[18] Carvalho Filho C.D., Honório S.L., Gil J.M. (2006). Qualidade pós-colheita de cerejas cv. Ambrunés utilizando coberturas comestíveis. Rev. Bras. Frutic..

[19] Chitarra M.I.F., Chitarra A.B. (1990). Pós-Colheita de Frutos e Hortaliças: Fisiologia e, Pós-colheita de Frutos e Hortaliças: Fisiologia e Manuseio.

[20] Schwartz E., Fachinello J.C., Barbieri R.L., da Silva J.B. (2010). Avaliação de populações de Butia capitata de Santa Vitória do Palmar. Rev. Bras. Frutic..

[21] Santas J., Carbó R., Gordon M., Almajano M. (2008). Comparison of the antioxidant activity of two Spanish onion varieties. Food Chem..

[22] Lv S., Taha A., Hu H., Lu Q., Pan S. (2019). Effects of Ultrasonic-Assisted Extraction on the Physicochemical Properties of Different Walnut Proteins. Molecules.

[23] Bouhafsoun A., Yilmaz M.A., Boukeloua A., Temel H., Harche M.K. (2018). Simultaneous quantification of phenolic acids and flavonoids in *Chamaerops humilis* L. using LC–ESI-MS/MS. Food Sci. Technol..

[24] Estrada A., Katselis G.S., Laarveld B., Barl B. (2000). Isolation and evaluation of immunological adjuvant activities of saponins from *Polygala senega* L.. Comp. Immunol. Microbiol. Infect. Dis..

[25] Just M.J., Recio M.C., Giner R.M., Cuéllar M.J., Máñez S., Bilia A.R., Ríos J.-L. (1998). Anti-Inflammatory Activity of Unusual Lupane Saponins from *Bupleurum fruticescens*. Planta Med..

[26] El Cadi H., El Bouzidi H., Selama G., El Cadi A., Ramdan B., Oulad El Majdoub Y., Alibrando F., Dugo P., Mondello L., Fakih Lanjri A. (2020). Physico-Chemical and Phytochemical Characterization of Moroccan Wild Jujube “*Zizyphus lotus* (L.)” Fruit Crude Extract and Fractions. Molecules.

[27] Belhaoues S., Amri S., Bensouilah M., Seridi R. (2017). Antioxidant, antibacterial activities and phenolic content of organic frac-tions obtained from *Chamaerops humilis* L. leaf and fruit. Int. J. Biosci..

[28] Khoudali S., Benmessaoudleft D., Essaqui A., Zertoubi M., Mohammed A., Benaissa B. (2014). Study of antioxidant activity and anticorrosion action of the methanol extract of dwarf palm leaves (*Chamaerops humilis* L.) from morocco. J. Mater. Environ. Sci..

[29] Coelho J.P., Veiga J.G., Elvas-Leitao R., Brigas A.F., Dias A.M., Oliveira M.C. Composition and in vitro antioxidants ac-tivity of *Chamaerops humilis* L.. Proceedings of the 2017 IEEE 5th Portuguese Meeting on Bioengineering (ENBENG).

[30] Chung-Weng P., Abd Malek S.N., Ibrahim H., Wahab N.A. (2011). Antioxidant properties of crude and fractionated extracts of Alpinia mutica rhizomes and their total phenolic content. Afr. J. Pharm. Pharmacol..

[31] Mokbli S., Sbihi H.M., Nehdi I.A., Romdhani-Younes M., Tan C.P., Al-Resayes S.I. (2018). Characteristics of *Chamaerops humilis* L. var. humilis seed oil and study of the oxidative stability by blending with soybean oil. J. Food Sci. Technol..

[32] Giovino A., Marino P., Domina G., Rapisarda P., Rizza G., Saia S. (2014). Fatty acid composition of the seed lipids of *Chamaerops humilis* L. natural populations and its relation with the environment. Plant Biosyst. Int. J. Deal. All Asp. Plant Biol..

[33] Khallouki F., Ricarte I., Breuer A., Owen R.W. (2018). Characterization of phenolic compounds in mature Moroccan Medjool date palm fruits (*Phoenix dactylifera*) by HPLC–PDA-ESI-MS. J. Food Comp. Anal..

[34] Ma C., Dunshea F.R., Suleria H.A.R. (2019). LC-ESI-QTOF/MS Characterization of Phenolic Compounds in Palm Fruits (Jelly and Fishtail Palm) and Their Potential Antioxidant Activities. Antioxidants.

[35] Singleton V., Rossi J. (1965). Colorimetry of Total Phenolic Compounds with Phosphomolybdic-Phosphotungstic Acid Reagents. Am. J. Enol. Vitic..

[36] Zhishen J., Mengcheng T., Jianming W. (1999). The determination of flavonoid contents in mulberry and their scavenging effects on superoxide radicals. Food Chem..

[37] Julkunen-Tiitto R. (1985). Phenolic constituents in the leaves of northern willows: Methods for the analysis of certain phenolics. J. Agric. Food Chem..

[38] Braca A., Sortino C., Politi M., Morelli I., Mendez J. (2002). Antioxidant activity of flavonoids from *Licania licaniaeflora*. J. Ethnopharmacol..

